# The effectiveness of simple heuristic features in sensor orientation and placement problems in human activity recognition using a single smartphone accelerometer

**DOI:** 10.1186/s12938-024-01213-3

**Published:** 2024-02-17

**Authors:** Arnab Barua, Xianta Jiang, Daniel Fuller

**Affiliations:** 1 Department of Computer Science, Faculty of Science, Memorial University of Newfoundland, St. John′s, A1B 1V6 Canada; 2 Department of Community Health and Epidemiology, University of Saskatchewan, Health Science Building, 107 Wiggins Road, Saskatoon, SK S7N 5E5 Canada

**Keywords:** 1D-CNN–LSTM, Metabolic Equivalent of Tasks, Accelerometer sensor, Human activity recognition, Sensor orientation, Sensor placement

## Abstract

**Background:**

Human activity Recognition (HAR) using smartphone sensors suffers from two major problems: sensor orientation and placement. Sensor orientation and sensor placement problems refer to the variation in sensor signal for a particular activity due to sensors’ altering orientation and placement. Extracting orientation and position invariant features from raw sensor signals is a simple solution for tackling these problems. Using few heuristic features rather than numerous time-domain and frequency-domain features offers more simplicity in this approach. The heuristic features are features which have very minimal effects of sensor orientation and placement. In this study, we evaluated the effectiveness of four simple heuristic features in solving the sensor orientation and placement problems using a 1D-CNN–LSTM model for a data set consisting of over 12 million samples.

**Methods:**

We accumulated data from 42 participants for six common daily activities: Lying, Sitting, Walking, and Running at 3-Metabolic Equivalent of Tasks (METs), 5-METs and 7-METs from a single accelerometer sensor of a smartphone. We conducted our study for three smartphone positions: Pocket, Backpack and Hand. We extracted simple heuristic features from the accelerometer data and used them to train and test a 1D-CNN–LSTM model to evaluate their effectiveness in solving sensor orientation and placement problems.

**Results:**

We performed intra-position and inter-position evaluations. In intra-position evaluation, we trained and tested the model using data from the same smartphone position, whereas, in inter-position evaluation, the training and test data was from different smartphone positions. For intra-position evaluation, we acquired 70–73% accuracy; for inter-position cases, the accuracies ranged between 59 and 69%. Moreover, we performed participant-specific and activity-specific analyses.

**Conclusions:**

We found that the simple heuristic features are considerably effective in solving orientation problems. With further development, such as fusing the heuristic features with other methods that eliminate placement issues, we can also achieve a better result than the outcome we achieved using the heuristic features for the sensor placement problem. In addition, we found the heuristic features to be more effective in recognizing high-intensity activities.

## Background

Human activity recognition (HAR) is the process of enabling computers to recognize human activities by analyzing patterns in different data types, including sensor data, images, and videos. Research on HAR is important as it is the principal method for accomplishing applications, such as identifying risk factors regarding depression [1], diabetes [2], health condition surveillance [3, 4], eldercare [5], sports performance analysis [6], and abnormal activity identification [7]. Since HAR is the primary foundation for the successful implementation of many applications, researchers are trying to overcome the challenges which cause inaccuracy in HAR. Sensor data is one of the most reliable and popular data types used in HAR. Sensor data includes data from accelerometers, gyroscopes, and magnetometers [8–10]. Studies have used these sensors in divergent ways to accumulate data for HAR. Some researchers attached the sensors separately to different body parts [11–13], and some used sensors embedded in smartphones [14–18] or smartwatches [19–21]. Among these different types of sensory devices and placements, smartphones are efficient, feasible and beneficial to HAR research, because they address a number of advantages, including applicability to a large population. Almost every smartphone contains an accelerometer and a gyroscope sensor. Data from both of these sensors are capable of distinguishing different human activities, which means they are feasible for HAR applications. Smartphones are also an inseparable part of daily human life. As a result, researchers emphasized improving the HAR using smartphone sensors by adapting various techniques to diminish the difficulties posed by smartphones in HAR.

**Fig. 1:**
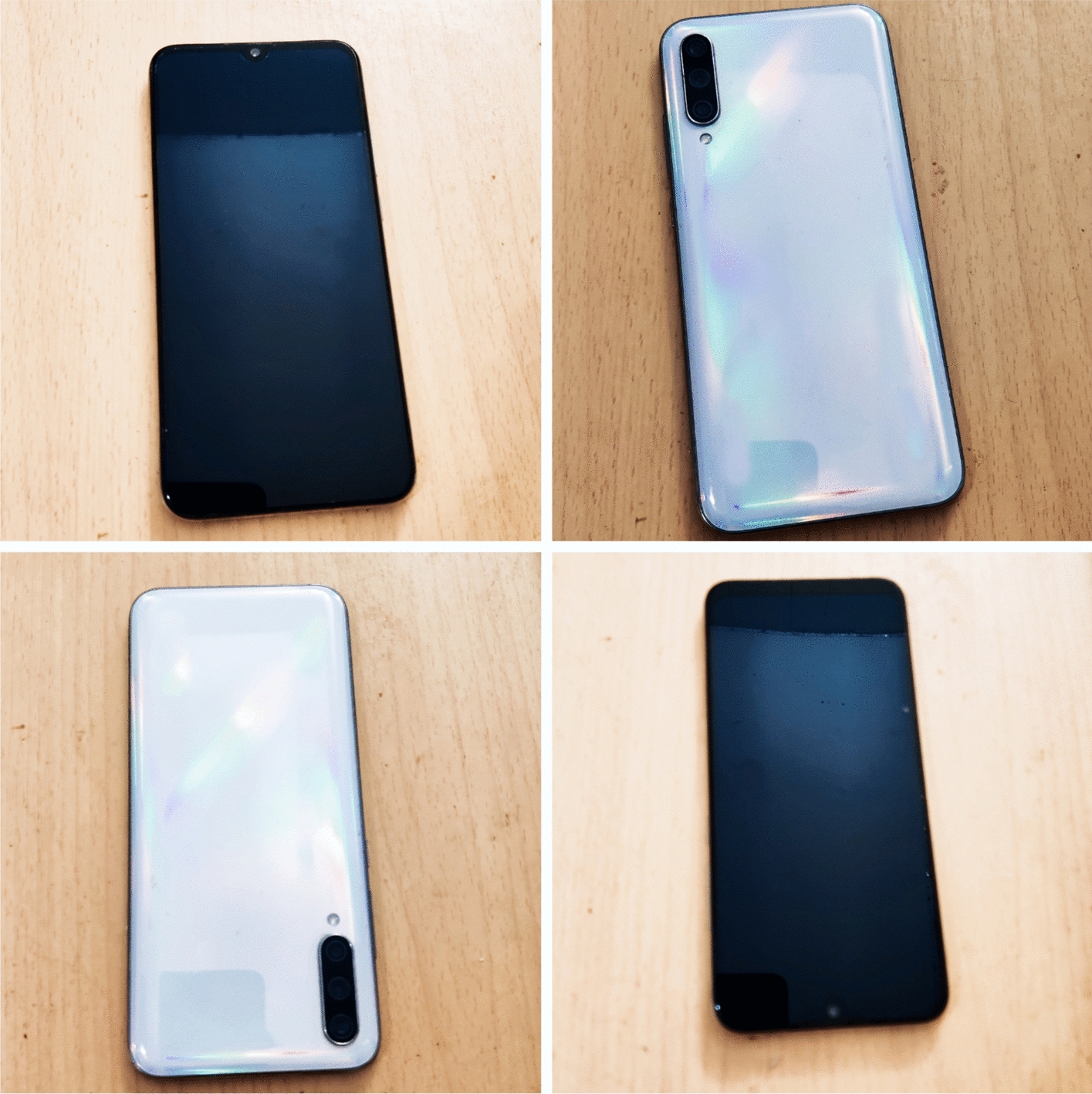
Possible orientations for a smartphone in a particular placement

Advancing the HAR process using smartphone sensors requires the researchers to overcome some significant challenges related to sensor orientation, sensor placement, and algorithm choice. The sensor orientation problem is one of the most concerning problems faced when using smartphones in HAR, as a smartphone can be kept in different orientations, as depicted in Fig. 1. A user can keep the smartphone in any orientation and perform different activities. When two different users perform the same activity while keeping the smartphone in different orientations, the sensor data becomes different, making it hard for HAR methods to identify the sensor data as the same activity. Many studies have proposed different methods to deal with the sensor orientation problem of the smartphone in HAR. Researchers also had to propose various approaches to diminish the sensor placement problems [22, 23]. The sensor placement problem happens as smartphone users tend to keep their smartphones in different body locations, including backpacks, hands, or pockets. The smartphone sensors, particularly the accelerometer and gyroscope sensors, generate non-identical patterns for similar activity if the smartphone is kept in non-identical locations. For dealing with the sensor orientation and placement problem, researchers generally try to extract features with no orientation or placement effect that could generate substantially different sensor patterns for different activities. For example, [24] used extracted features in their proposed activity recognition process, where they included data from four different body locations (coat pocket, hand, trouser pocket and bag) and for five human activities (going upstairs, going downstairs, walking, standing and running). They started by extracting horizontal and vertical acceleration data from a raw accelerometer to diminish the influence of device orientation. Later, they extracted eight features from the raw gyroscope signal and separated horizontal and vertical accelerations to develop a position identification system. Finally, they performed feature selection, and using this position recognition system, they conducted some data adjustments to the selected features, which were later used in their activity recognition process. They achieved an accuracy of 91.27% using a Support Vector Machine (SVM) with a 4-fold cross-validation technique. Chen and Shen [25] extracted 89 time and frequency domain features from smartphones’ accelerometers and gyroscope sensors to make the activity recognition process orientation invariant and position independent. They then performed feature selection and feature normalization on the extracted features. Using these features, they evaluated the performance of three classifiers, K-Nearest Neighbours (KNN), Random Forest (RF), and SVM in recognition of five human activities (descending stairs, ascending stairs, walking, jogging and jumping) for five non-identical smartphone locations (right upper arm, right hand, right jacket pocket, right trousers pocket and waist). They considered different validation procedures named one-to-one, all-to-one and rest-to-one and compared the performance of the classifiers for different validation procedures. Yurtman and Barshan [26] extracted 9 heuristic features from the data of different sensors available in five public data sets, which they claimed to be free of the influence of sensor orientation. They evaluated the performance of these 9 features in HAR using four machine learning algorithms and found them compelling enough to diminish the orientational effects. Along with these studies, many other studies extracted features to solve the sensor orientation and location dependency problem [27–30]. However, along with feature extraction process, coordinate transformation is a promising method to address the sensor orientation and location dependency problem.

In the coordinate transformation approach, first, a global reference coordinate system is discovered, and then all the signals are projected to that reference system. Guo et al. [31] used a coordination transformation approach on the gyroscope signal from a smartphone fused with a motif discovery algorithm to find activity patterns and then developed a Vector Space Model for classification purposes. They used their approach on a data set containing smartphone signals from four different body positions (left upper arm, the shirt pocket, the trousers front pocket, and the behind trouser pocket) and four different orientations and performed cross-orientation and cross-placement validation. Chen et al. [32] also performed coordination transformation by calculating quaternion to transform the linear acceleration signal from the device-coordinate system to the earth-coordinate system. Following, they extracted the first two principal components from the transformed acceleration signal to eliminate the direction effect for different activities. In addition, they extracted time and frequency domain features to make their approach more reliable and accurate. To validate their method, they collected data from a smartphone placed in three different positions (pants’ pocket, shirt’s pocket and backpack) and three different orientations. They performed a leave-one-orientation-out cross-validation technique using an Online SVM algorithm and compared results for different orientations, placements and participants. Ustev et al. [33] also performed coordinate transformation and feature extraction using an accelerometer, gyroscope, and magnetic sensor to eliminate the orientational effect of the smartphone sensor. They evaluated their method for two smartphone orientations (vertical and horizontal) placed in trouser pockets. They achieved 97% accuracy in recognizing five human activities using a KNN classifier. A summary of the studies discussed above is presented in Table 1. In brief, several studies and methods have been used to address the sensor orientation and placement problem. However, the classification algorithm also plays an important role in HAR classification accuracy, in particular deep learning algorithms are potentially promising given a sufficiently large data set.

**Table 1:** Summary of the previous studies which used feature extraction and coordinate transformation methods

Method	Sensor types	Sensor positions	Classifier	References
Feature extraction	Accelerometer and gyroscope	Coat pocket, hand, trouser pocket and bag	SVM	[24]
Feature extraction	Accelerometer and gyroscope	Right upper arm, right hand, right jacket pocket, right trousers pocket and waist	KNN, RF, SVM	[25]
Feature extraction	Accelerometer, gyroscope and magnetometer	Multiple positions from 5 data sets	Bayesian decision making (BDM), KNN, SVM, Artificial Neural Network (ANN)	[26]
Coordination transformation	Accelerometer and gyroscope	Left upper arm, the shirt pocket, the trousers front pocket, and the behind trouser pocket	Motif-based classification system	[31]
Coordination transformation and feature extraction	Accelerometer	Pants’ pocket, shirt’s pocket and backpack	Online SVM	[32]
Coordination transformation and feature extraction	Accelerometer, gyroscope and magnetic field	Trouser pockets	KNN	[33]

Studies have evaluated the performance of divergent machine learning algorithms in HAR and provided comparisons to decide the most suitable classifiers to use. Early classification studies used simple machine learning classifiers, such as SVM [27, 34–36], RF [37–39], KNN [40, 41], and decision trees [42, 43]. These were employed because of their low complexity and resource-efficient nature. However, the advancement of computational resources enabled the usage of deep learning algorithms, such as artificial neural networks (ANN), convolutional neural networks (CNN), recurrent neural networks (RNN), long–short-term memory (LSTM), and gaited recurrent units (GRU). These deep learning algorithms offer additional advantages in HAR, especially CNN and LSTM because of CNN’s automated feature extraction capability and LSTM’s ability to persist older information from time series data. Yang et al. [44] used CNN’s ability of automatic feature learning and found it to outperform four conventional machine learning algorithms in recognizing 18 human activities and 12 hand gestures. They also concluded that CNN was suitable for online HAR. Zeng et al. [45] also exploited the feature extraction ability of CNN for HAR on three public data sets (Opportunity, Skoda and Actitracker) and acquired an accuracy of 88.19%, 76.83%, and 96.88% on Skoda, Opportunity, and Antitracker, respectively, using the CNN-based model. Xu and Qiu [46] evaluated the feature extraction capability of CNN in recognizing six daily human activities (sitting, standing, walking, jogging, upstairs, and downstairs) using accelerometer signals. They achieved an accuracy of 94.2%, which outperformed traditional machine learning algorithms, such as Decision Trees (J48) and SVM. There are also other studies that used CNN as their final classification model, along with their early data pre-processing layer, to enhance the recognition rate of human activities [47–49]. Along with CNN, another deep neural network variation called RNN is being widely used in HAR [50–52]. RNN has few variations of itself, and among them, LSTM is useful in HAR, especially when studies combine the information-persistence ability of LSTM with the feature extraction capability of CNN. Xia et al. [53] utilized the combination of CNN and LSTM, also called CNN–LSTM, to evaluate its performance in HAR on two data sets (iSPL and UCI HAR). They acquired 99.06% and 92.13% accuracy on iSPL and UCI HAR data sets, respectively. Mekruksavanich and Jitpattanakul [54] also employed the CNN–LSTM model for HAR using data from smartwatch sensors from 44 subjects performing 18 activities. They achieved an accuracy of 96.20% using CNN–LSTM, which was better than the performance of CNN and LSTM when the models were used separately. Mekruksavanich and Jitpattanakul [55] proposed a 4-layered CNN–LSTM model and evaluated its performance using the UCI HAR data set. They found that the CNN–LSTM hybrid model can outperform Vanilla LSTM network, 2-Stacked LSTM network, 3-Stacked LSTM network achieving an accuracy of 99.39% using a 10-fold cross-validation technique. Many other researchers have used CNN–LSTM in HAR to utilize its capabilities of feature extraction and preserving temporal dependencies [56–59]. There has been considerable research for HAR which proposed divergent techniques to solve the major challenges, including sensor orientation, sensor placement, and algorithm choice.

In this study, we contributed to this field by evaluating the performance of previously introduced heuristic features [26] using our data set in intra-position (i.e., senor orientation problem) and inter-position (i.e., sensor placement problem) scenarios using a 1D-CNN–LSTM model. In the original study [26], the researchers introduced heuristic features to tackle the sensor orientation problem. However, they evaluated the performance of those heuristic features by synthetically introducing orientation in the data set. In our study, we assessed the performance of these features in solving the orientation problem for three different positions, where the sensor orientations were ensured during the data accumulation process. Moreover, we assessed the performance of those heuristic features in solving the sensor placement problem. By doing this, we wanted to inspect if the heuristic features alone can solve the sensor placement problem. In addition, only a few studies adopted the Leave-*N*-Subject-Out Cross-Validation approach and did it for a considerably small-scale data set. In our study, we adopted the Leave-*N*-Subject-Out Cross-Validation approach for a data set accumulated from 42 subjects and consisting of over 12 million samples. To be precise, we worked on the following contributions in this study,

- We evaluated the effectiveness of previously proposed sensor invariant features’ [26] performance in the case of sensor orientation problems in HAR for a large-scale data set, where the sensor orientations were practically introduced. Previously, the performance of the heuristic features was evaluated using data, where the orientations were introduced synthetically (intra-position evaluation)
- No study in the past evaluated the effectiveness of the heuristic features in solving sensor placement problems. In our study, we assessed the performance of heuristic features in tackling the sensor placement problem in HAR (inter-position evaluation)
- We analyzed the performance of the proposed approach in HAR using a Leave-10-Subject-Out Cross-Validation technique for a vast data set containing enormous variations. Previously, most of the studies used Leave-1-Subject-Out Cross-Validation and used comparatively small data sets. Our employed Leave-10-Subject-Out Cross-Validation technique is more challenging for the HAR system than the Leave-1-Subject-Out Cross-Validation technique.
- We analyzed the performance of the proposed architecture for six activities with varying intensities (Lying, Sitting, Walking, Running at 3 METs, Running at 5 METs, and Running at 7 METs). We wanted to forge the whole system as practically as possible by introducing sensor orientation, placement problems, and activities with varying intensities.

**Fig. 2:**
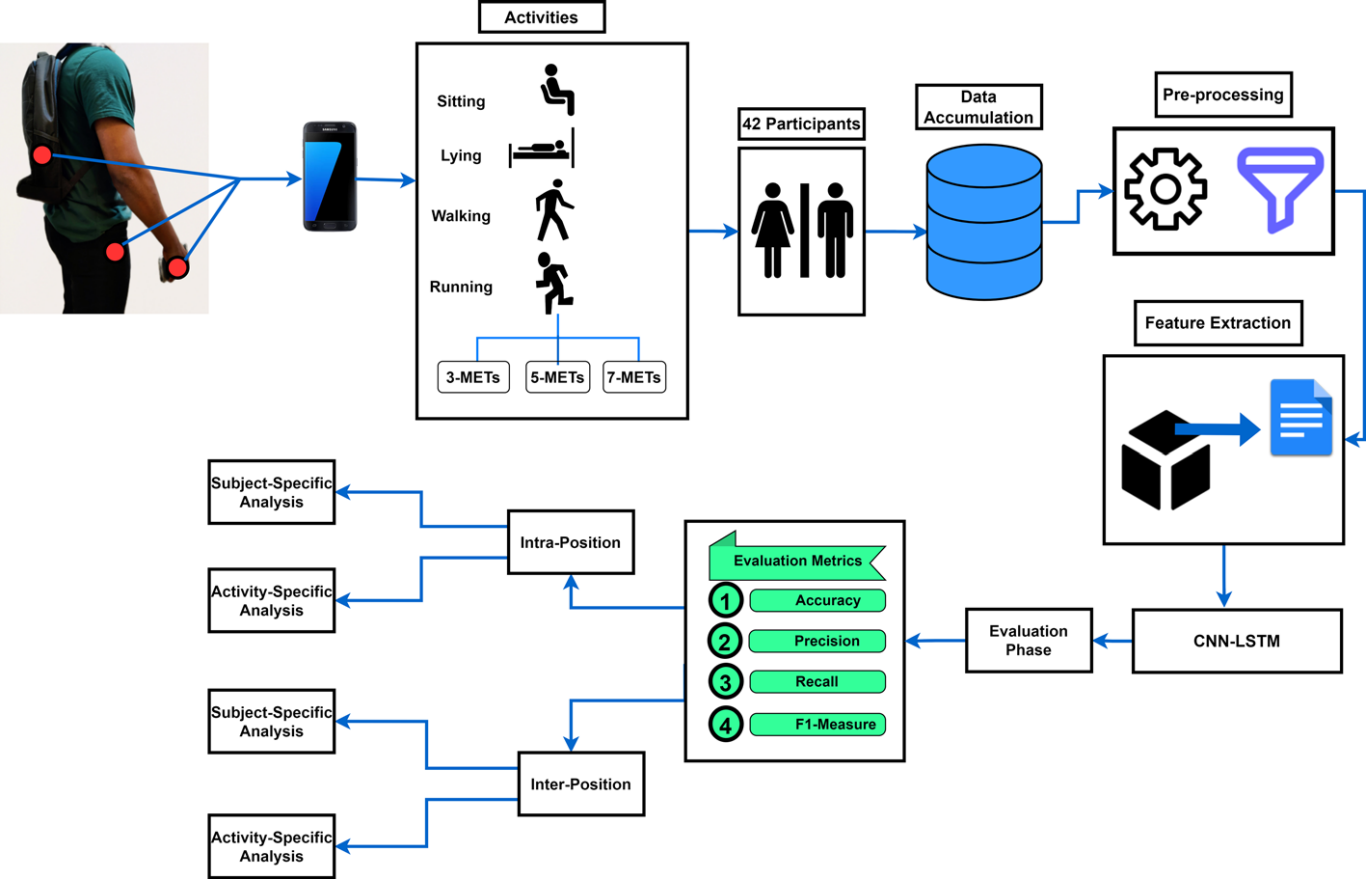
Overall workflow diagram of our study

The rest of the paper is arranged as follows. “Results” section describes activity-specific and participant-specific results for both intra-position and inter-position scenarios. “Discussion” section discusses our findings, and we conclude our study in “Conclusion” section. “Methods” section introduces the materials and methods, where we discuss the data accumulation procedures, data pre-processing and feature extraction approach, the architecture of the models, and their workflow. The entire study procedure is depicted in Fig. 2.

## Results

We used data from three positions, and the users had the freedom to keep the smartphone in each position at any orientation. We used the four most common evaluation metrics for multi-class classification studies: Accuracy [60], Precision [60], Recall [60], and F1-Score [61]. Accuracy is the most suitable metric to present a classification model’s overall performance. The other three metrics are well-suited to describe the model’s performance for the class-specific scenario.

### Results for intra-position scenario

In intra-position evaluation, we first analyzed the model’s overall performance for each position. Following, we performed participant-specific and activity-specific analyses.

#### Overall result

**Fig. 3:**
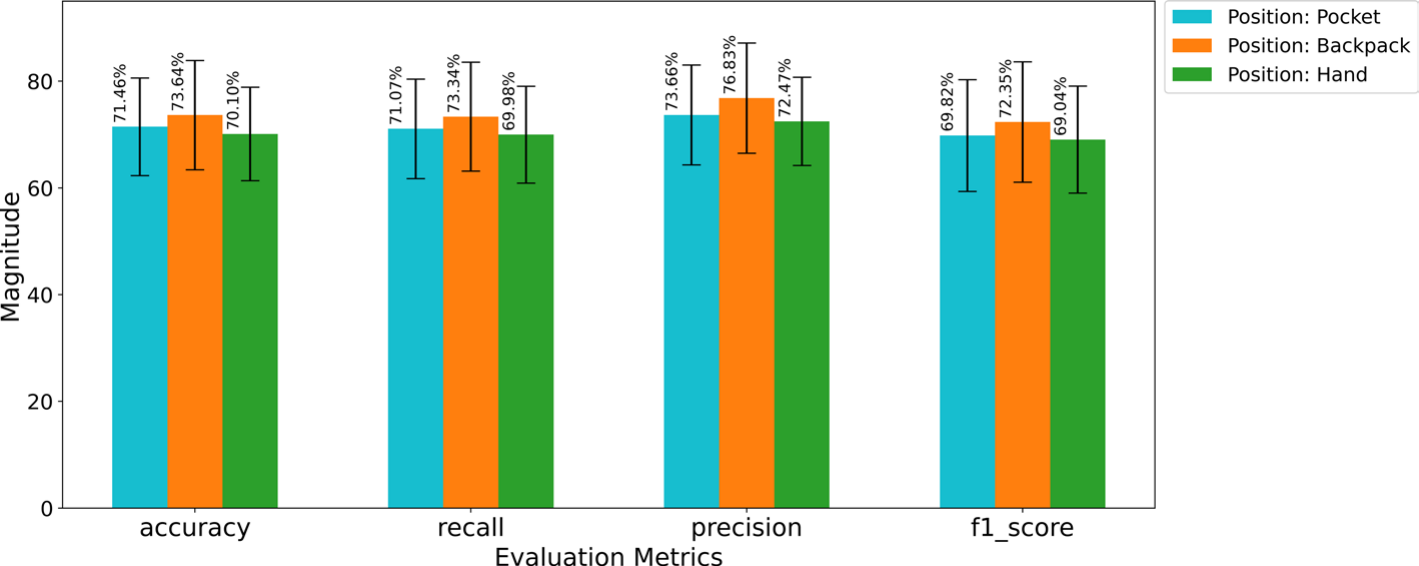
Bar plots with error bars showing averaged evaluation metrics for the intra-position scenario

For the intra-position case, the model was trained and tested using the heuristic features corresponding to the same position. We computed results using the Leave-10-Subject-Out Cross-Validation procedure and averaged the test results. The average accuracy, recall, precision and F1-Score for each position are depicted in Fig. 3.

In the intra-position scenario, we achieved the highest result for the position backpack for every evaluation metric. We recorded 73.64% accuracy, 73.34% recall, 76.83% precision and 72.35% F1-Score for the position backpack. We recorded the second-best results for the position pocket. For the position pocket, the accuracy, recall, precision and F1-Score were 71.46%, 71.07%, 73.66% and 69.82%, respectively. Although the results were lower for the position hand among all the positions, they were not much lower than those for the position pocket. We recorded 70.10% accuracy, 69.98% recall, 72.47% precision and 69.04% F1-Score for the position hand. We hypothesize that we achieved better results for the backpack position, because the smartphone was more stable in the backpack than in the other positions. For activities with high intensities, such as walking or running, the hand frequently moved with the body, which allowed additional variations for the values from the accelerator sensor of the smartphone. Consequently, the values for the heuristic features were affected for the position hand, and the results became lower. If we observe the overall results, the evaluation metrics ranged between 69% and 74% for all the positions. We cannot consider it the best result compared to the previously conducted studies. Still, considering the number of participants, the volume of the data set and the number of sensors, the results seem promising. The accuracies were over 70% for all the positions, which means that the 1D-CNN–LSTM model performed decently as a classification model. The average precision and recall were promising, indicating that our model tried to keep the number of false predictions lower and true predictions higher for each activity class. However, these two metrics will be more meaningful when we observe their value for the activity-specific scenario. The satisfactory F1-Score meant that the 1D-CNN–LSTM model tried to maintain a balanced trade-off between precision and recall.

#### Participant-specific scenario

**Fig. 4:**
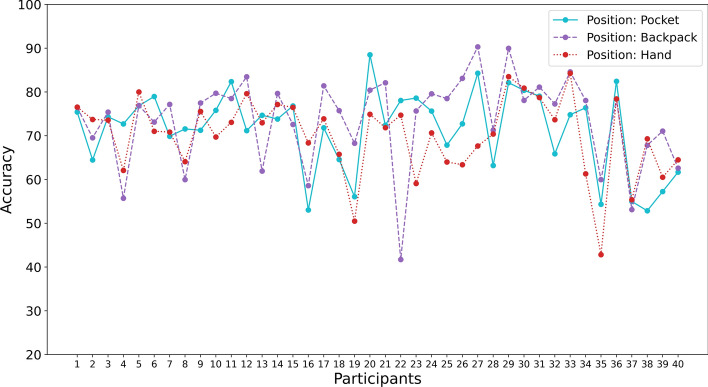
Line plot showing accuracies for all participants at every position in the intra-position scenario

We only considered accuracy as a summary metric of model performance for the participant-specific result analysis in the intra-position scenario. We wanted to observe how consistent the model’s performance was for each subject. The accuracy of each participant for each smartphone location is depicted using a line plot in Fig. 4.

For the position pocket, we achieved the highest accuracy of 88.49% for Participant 20. For most participants, the accuracy ranged from 60% to 80%. However, we recorded inferior accuracy in the case of some participants, such as participants 16, 35, 37 and 38. For the position backpack, we recorded the highest accuracy of 90.29% for Participant 27. The accuracy range for most participants was the same as we observed in the pocket case. We also observed inferior performance from the model for some participants, such as participants 4, 16, 22 and 37. For position hand, the highest accuracy was 84.25% for Participant 33. The overall accuracy range was the same as we observed for other positions. Again, the model rendered insufficient accuracy for participants, such as 19, 25 and 37. Considering the overall pictures, for the intra-position scenario, the performance of heuristic features can be regarded as sufficient and propitious. Some participants, including 16 and 37, consistently had low accuracy across all intra-position scenarios. It is somewhat unclear why this is the case, but likely, the result is due to noise in the raw data.

#### Activity-specific scenario

**Fig. 5:**
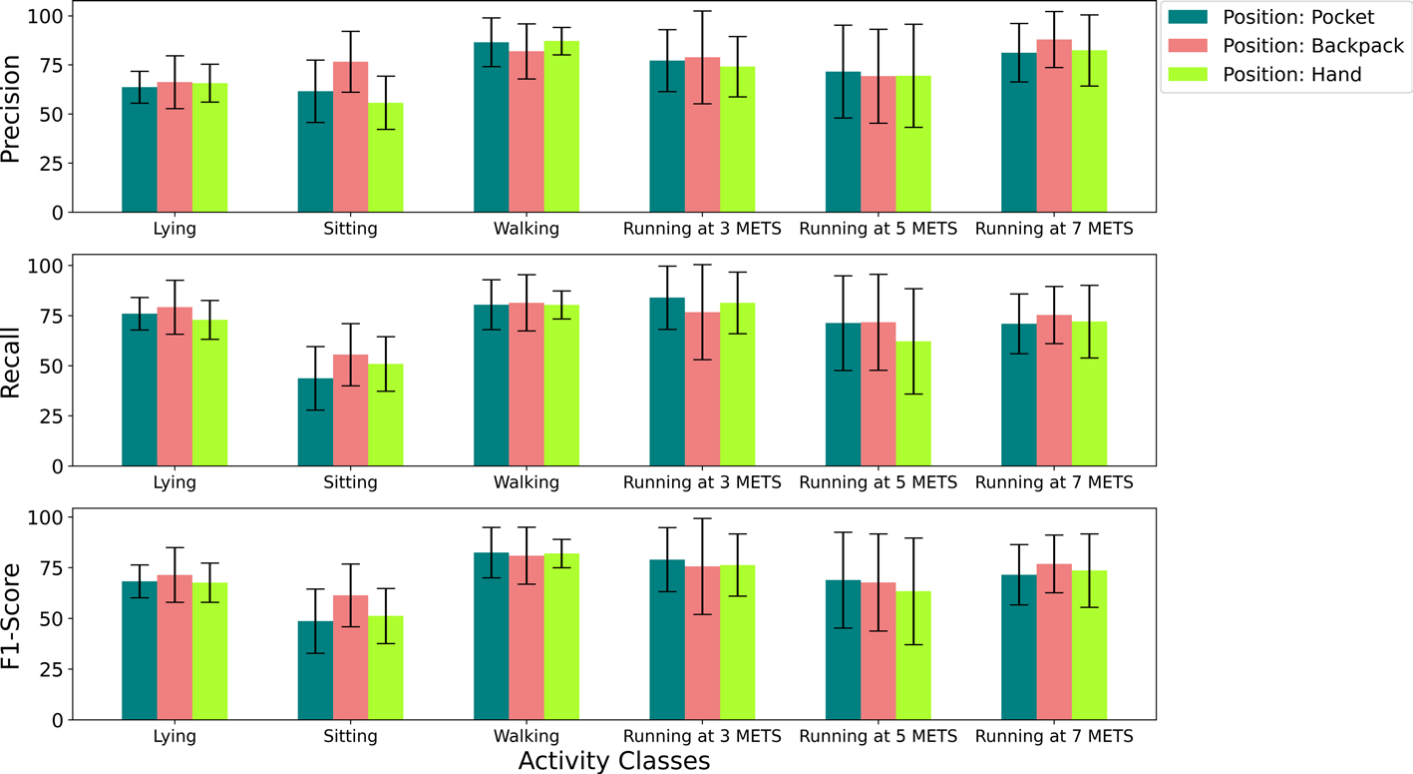
Bar plot showing activity-specific results with error bars for the intra-position scenario

We also analyzed the result of the intra-position scenario for the activity-specific case. For this analysis, we considered the evaluation metrics such as recall, precision and F1-Score to demonstrate how the heuristic features performed with the help of the 1D-CNN–LSTM model for each activity class. The results for the activity-specific case are depicted in Fig. 5.

First, we will discuss the values of evaluation metrics for the pocket position. We recorded the highest precision of 86.58% for the activity “Walking”. We generally expect a model to generate high precision for all the classes. Our 1D-CNN–LSTM model generated high precision for high-intensity activities such as Walking and Running at 3, 5 and 7 METs for the data of the position pocket. However, the precision for low-intensity activities such as Sitting (61.61%) and Lying (63.70%) was low. This is a well-known result, because sensor signals tend to be small for low-intensity activities; therefore, models misclassify these activities. For the pocket position, all activity classes, except Sitting, had a model-generated recall greater than 70%. We recorded the highest recall of 83.96% for the activity Running at 5 METs. We also expect high recall from a classification model along with high precision. However, our model for the pocket location generated very poor recall (43.76%) for the low-intensity activity of Sitting. The F1-Score in our classification model is a critical metric, because it explains how well-balanced our model is for precision and recall. For the pocket position, the F1-Score was promising for all the activities except for Sitting. For the activity class Sitting, the F1-Score was only 48.65%. The F1-Score was expected to be low for the activity Sitting as we experienced low precision and recall for that same activity. We acquired the highest F1-Score for the activity Walking (82.46%).

For the backpack position, we recorded the highest precision of 87.98% for activity Running at 7 METs. The precision was lower for activities, such as Lying (66.26%) and Running at 5 METs (69.31%). The precision for the activity Sitting was poor for the pocket position; however, for the position backpack, it was improved (76.60%). The recall for the backpack position was similar to the pocket position. We recorded the lowest recall of 55.55% for the activity Sitting. The highest recall was found for the activity of Walking (81.42%). The recall ranged between 70% and 80% for all other activities. The scenario for F1-Score for the position backpack was similar to the position pocket. The lowest F1-Score was recorded for the activity Sitting (61.41%), and the highest F1-Score was recorded for Walking (80.95%). Considering the evaluation metrics for the position backpack, our model seemed to struggle to identify the activity Sitting correctly.

We expected the evaluation metrics for the hand position to be poorer than those for the other positions. This is because, as we mentioned before, the continuous movement of the hand during high-intensity activities causes extensive variations in the data collected from the accelerometer sensor. The precision values for the hand position had a pattern similar to that observed for the pocket position. We recorded the highest and lowest precision for the Walking (87.17%) and Sitting (55.76%) activities, respectively. Regarding recall for the hand position, the scenario was the same as we observed for the pocket position. The recall was highest for the activity Running at 3 METs (81.41%) and lowest for the activity Sitting (50.95%). The recall was poor for the activity Running at 5 METs. The lowest F1-Score for the hand position was recorded for the activity Sitting (51.25%), and the highest F1-Score was for the activity Walking (81.99%). Considering the precision, recall, and F1-Score for all the positions, we found that the model struggled to recognize the activity Sitting for all three positions. For other activities, the model performed well using the heuristic features, especially for the activity Walking.

### Results for inter-position scenario

In the case of the inter-position scenario, we performed the same analysis. We will start by discussing the overall results. Following, we will describe the participant-specific and activity-specific results.

#### Overall results

We trained our model using heuristic features extracted from the raw accelerometer data from one smartphone placement and tested the model’s performance using the heuristic features extracted from the raw accelerometer data of a different smartphone placement. We averaged the evaluation metrics over all the iterations of the validation procedure to calculate the final overall results. The results are shown in Table 2. The highest accuracy was for the backpack position when the model was trained using the data from the hand position. We recorded 68.66% accuracy, 69.95% precision, 67.07% recall and 64.77% F1-Score in this case. The lowest accuracy result was recorded for the data from the hand position when the model was trained using data from the backpack position. The accuracy and F1-Score were below 60% in this case. When the model was trained using data from the pocket position and tested using data from the backpack position, we acquired results that were almost similar to the case, where the model was trained using the data from hand and tested using the data from the backpack. For other cases, the metrics ranged between 62 and 66%.

**Table 2:** Averaged values of evaluation metrics for each inter-position case

Smartphone position for the training set	Smartphone position for test set	Accuracy (%)	Precision (%)	Recall (%)	F1-score (%)
Pocket	Backpack	67.80	69.56	66.93	65.00
	Hand	62.39	63.27	64.20	60.86
Backpack	Pocket	62.62	66.68	63.59	61.45
	Hand	59.03	61.64	60.60	58.23
Hand	Pocket	64.10	66.09	62.92	60.38
	Backpack	68.66	69.95	67.07	64.77

We expected to have poorer results in the case of inter-position evaluation, since the training data and test data were from different positions and different participants. The values for the evaluation metrics were below 70%. However, the result seems acceptable considering the simple heuristic features and data from a single accelerometer. The model seemed to perform the best when trained using the data from hand.

#### Participant-specific result

**Fig. 6:**
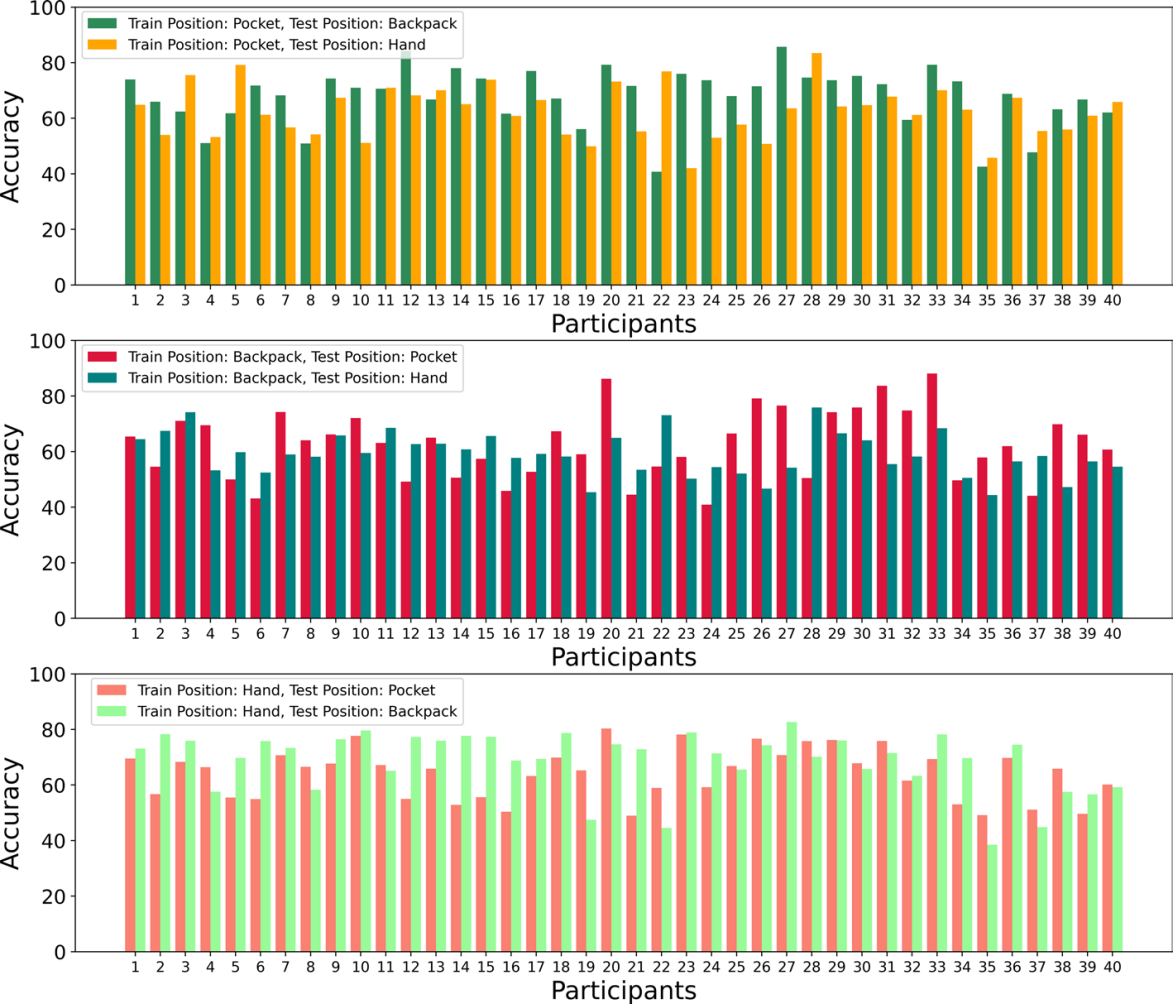
Participant-specific results for the inter-position scenario

We only considered accuracy as an evaluation metric for participant-specific evaluation. As mentioned before, the principal purpose of this analysis was to observe the number of participants for whom the model’s performance was poor. The analysis is depicted graphically in Fig. 6.

When the model was trained using data from the pocket position and tested using the data from other positions, most participants’ accuracies were above 60%. In addition, the accuracies were consistent for each participant for every position, i.e., for a particular participant, if the model performed well for the data from the hand position, the model performed well for the data from the backpack position. However, there were some exceptions; for instance, for participant 22, the accuracy of the data from the backpack was the lowest (40.71%), but the accuracy of the data from hand was 76.81%. For participant 23, the accuracy was 42.05% when the model was tested using data from the hand position, but for the same participant, the accuracy was 75.97% when tested using data from the backpack position.

When the model was trained using the data from the backpack, for some participants, the model performed very well when the test data was from the pocket. For example, for participants 20, 31 and 33, the accuracies were 86.22%, 83.69% and 88.13%, respectively. However, the scenario was not the same when the test data was from the hand position. The highest accuracy recorded when the test data was from the hand position was 75.91% for Participant 28. When the test data was from the hand position, the accuracy was below 47% for some participants, such as 19, 26 and 35. Similar accuracies were recorded for participants 6, 16, 21, 24 and 37 when the test data was from pocket.

For the final case, where the model was trained using data from hand and tested for two other positions, the results were better for most participants when the test data was from the backpack position. For most of the participants, the accuracies were about 70%. We recorded the highest accuracy of 82.67% for participant 27 when the training data was from the hand position, and test data was from the backpack position. Still, there were some participants, such as 19, 22, 35, and 37, for whom the accuracy was very low. When the test data was from the pocket position, we recorded the best accuracy (78.15%) for Participant 20. For most participants, accuracies were around 60%, except for some participants, such as 21, 35 and 39, where the accuracies were below 50%.

#### Activity-specific results

**Fig. 7:**
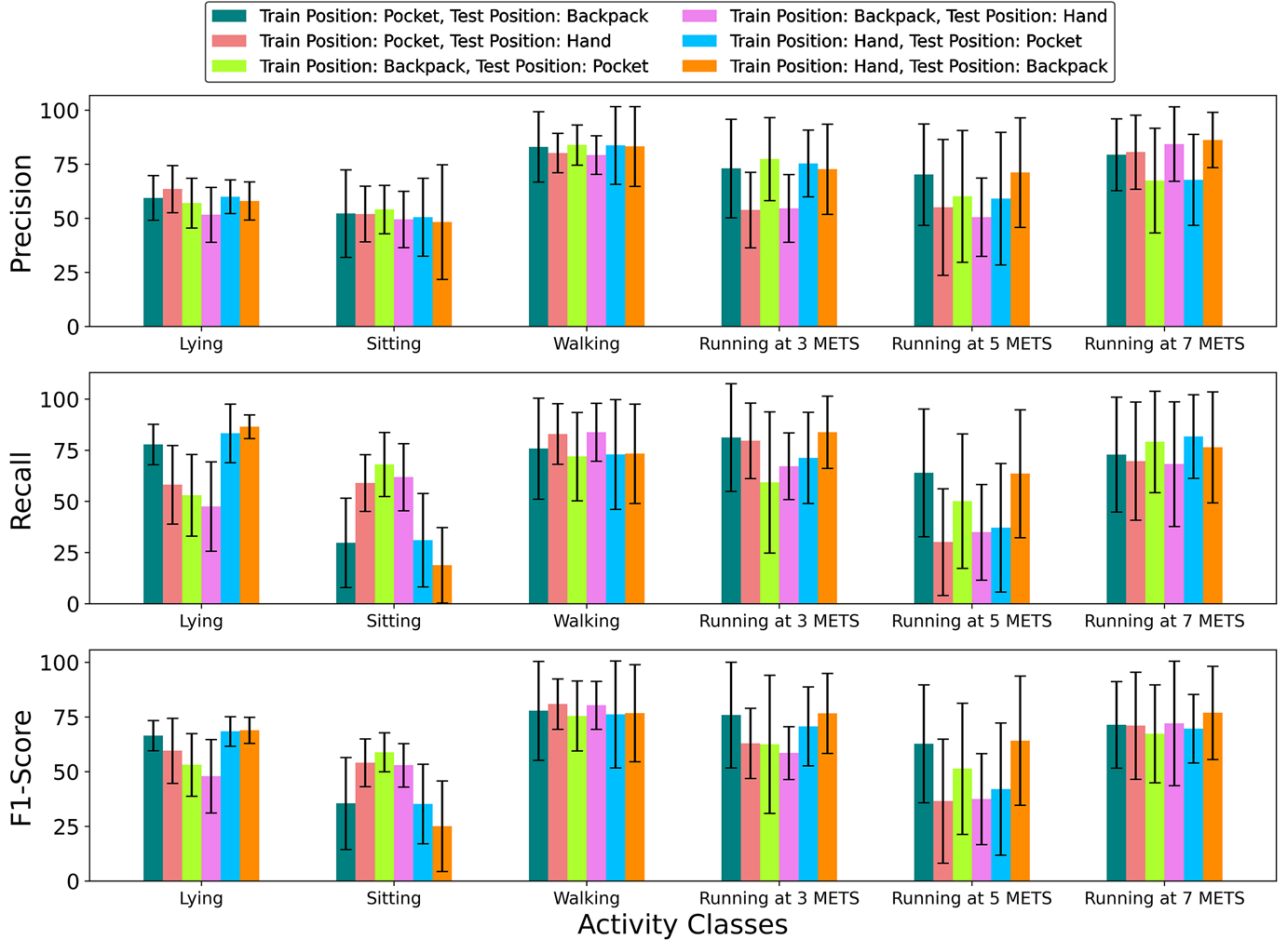
Activity-specific results for the inter-position scenario

For the activity-specific results, we will discuss each evaluation metric for all the cases individually. The evaluation metrics for each inter-position scenario for every activity class are depicted in Fig. 7.

For precision, we can see that the lowest values were found for the activity Sitting. For all the inter-position cases, the precision for the activity Sitting was around 50%. We encountered similar results for intra-position cases. The model found it challenging to identify the activity Sitting correctly in both inter-position and intra-position situations. The precision for the activity Walking was satisfactory for all the inter-position cases and was around 80%. For the activity Lying, we experienced precision ranging between 50% and 60%. This means the model mislabelled many samples from other high-intensity activities to Sitting and Lying. For the activity Running at 3 METs, the precision was lower than 60% for two cases, and in both cases, the test data was from the hand position. For other cases, the precision was around 70%. For the activity Running at 5 METs, the precision was approximately 70% when test data was from the backpack position. For other cases, the precision was around 60%. Finally, for the activity Running at 7 METs, the precision was approximately 80% except for two cases. The test data were from the pocket in both cases, and the precision was around 65%. Observing the precision for all activity classes, it is clear that the model had lower accuracy from low-intensity activities, which decreased overall precision.

Regarding the recall for all the inter-position cases, we recorded the poorest performance for two activity classes, Sitting and Running at 5 METs. For the activity Sitting, we recorded poor recall (between 20% and 30%) in three cases. In two of those three cases, the test data was from the backpack position, the other had data from the pocket position as test data, and the training data was from the hand position. In other cases, the recall was between 55 and 70%. Poor precision and recall for the activity Sitting means that the model mislabelled other activities as the activity Sitting and mislabelled many samples from Sitting activities to other activities. Although the precision was around 60% for all the cases of activity Lying, the recall was comparatively better and around 80% for three cases. In two of those three cases, the train data was from hand, and the other case had training data from the pocket position and test data from the backpack position. For Walking and Running at 3 METs, the recall was satisfactory and ranged between 70 and 80% for most inter-position cases. As mentioned before, the recall was poor for the activity Running at 5 METs. The recall (above 60%) was comparatively better for the activity Running at 5 METs only when the test data was from the backpack. For the activity Running at 7 METs, the recall was satisfactory. After observing the precision and recall, one significant finding was that the metrics were always better when the test data was from the backpack position.

In the case of F1-Score, the result was similar to the previous two metrics as it is a harmonic mean of recall and precision. The F1-Score was promising for the activity Walking, at approximately 80%, and Running at 7 METs, at about 70%, for all the cases. The F1-Score was low for the activities Sitting and Running at 5 METs. The low value of F1-Score for the activities Sitting and Running was expected as we experienced low precision and recall for those activities. For the activities Lying and Running at 3 METS, the F1-Score was better for three cases. Among those three cases, two cases had the data from the hand as train data, and in the other case, the test data was from the backpack, and the train data was from the pocket.

Considering all evaluation metrics for all the inter-position cases, we can conclude that the heuristic features with the 1D-CNN–LSTM model struggled with low-intensity activities in both intra-position and inter-position cases. Along with the low-intensity activities, we also found poor performance for the activity Running at 5 METs.

## Discussion

In our study, we wanted to explore the effectiveness of heuristic features in solving sensor orientation and sensor placement problems with the help of a 1D-CNN–LSTM model. We collected data from only a smartphone accelerometer sensor. We had 42 participants, and we followed the Leave-10-Subject-Out Cross-Validation approach. Our study had two types of analysis: intra-position (i.e., sensor orientation problem) and inter-position (i.e., sensor placement problem) analyses. In the intra-position scenario, we checked the effectiveness of the heuristic features on a data set, where the orientations were introduced during data collection. In the study [26], where the heuristic features were introduced, they evaluated the performance of the heuristic features on five public data sets, which they named A [62], B [63], C [64], D [65], and E [66]. These data sets were accumulated using sensors fixed at a constant orientation. Therefore, the data sets had orientation information for only one orientation. Their study claimed that the heuristic features removed the orientational information from the data set. Since the orientation information was removed, the data set simulated the scenario, where the orientation of the sensor did not matter anymore. However, their data set did not represent a practical scenario, where multiple orientations can be present. Since, in our case, different orientations of smartphone accelerometer were ensured, we were able to evaluate the performance of the heuristic features in a practical scenario. In addition, the data set on which [26] performed the evaluation had a low volume of data. They had the highest number of data windows for data set C (30 subjects); the number of data windows was 10,299, with a 50% overlapping ratio. In our case, we had around 4 million data windows for each position. For 3 positions, the total number of data windows was about 12 million. Moreover, we had 42 participants’ data, which offered more diversity for our data set. Their study followed the Leave-1-Subject-Out Cross-Validation and P-Fold Cross Validation approaches, whereas our Leave-10-Subject-Out Cross-Validation approach ensured a more practical test case, where 10 test participants offered completely unseen data to the model. In their study, three of their five data sets had more than one type of sensor. Data sets B and E only used a single accelerometer as we did. To be brief, our study protocol was more practical and simulated a real-life scenario, ensuring a more reliable evaluation of the heuristic features.

As mentioned earlier, the study [26] followed two validation approaches: P-Fold Cross Validation and Leave-1-Out Cross-Validation. The Leave-1-Out Cross Validation approach and our Leave-10-Out Cross-Validation approach tested the model using data from participants unseen by the model. They introduced 9 heuristic features and used these features by dividing them into 3 sets. The first set had the first 3 heuristic features, the second set had the first 6 features, and the third one had all 9 heuristic features. They evaluated the effectiveness of these features for 4 different classifiers: Bayesian Decision Making (BDM), K-Nearest Neighbour (KNN), Support Vector Machine (SVM) and Artificial Neural Network. In Table 3, we have tabulated the best result for each data set using the heuristic features, number of features used, name of the classifier, types of sensors, number of sensor units and the same information in our intra-position cases. We only included intra-position cases, because their study was conducted to solve sensor orientation problem for a fixed position.

**Table 3:** Accuracy comparison between the result obtained in [26] and our study

Data set	Types of sensors	Number of sensor units	Classifier	Number of features	Best accuracy (%)
A	Accelerometer, gyroscope, magnetometer	5	SVM	6	77.66
B	Accelerometer	4	SVM	3	86.92
C	Accelerometer, gyroscope	1	SVM	3	66.69
D	Accelerometer, gyroscope	1	SVM	3	50.62
E	Accelerometer	1	SVM	9	55.19
Our data set (pocket)	Accelerometer	1	1D-CNN-LSTM	4	71.46
Our data set (backpack)	Accelerometer	1	1D-CNN-LSTM	4	73.65
Our data set (hand)	Accelerometer	1	1D-CNN-LSTM	4	70.10

We found better accuracies for all the intra-position cases when compared with the accuracies they found for data sets C, D and E. Moreover, from their results, we can see that they found the best result for three of their data sets using only the first 3 heuristic features. Therefore, their study’s findings supported selecting the first four heuristic features based on feature importance. In addition, the satisfactory results we found for intra-position cases depict that the heuristic features effectively solve the orientation problem even for practical scenarios.

In our study, we also presented the performance of the heuristic features in participant-specific and activity-specific scenarios. In participant-specific cases, we found that, for most of the participants at each position, the accuracies were around 70%. There were some participants for which the models’ performance was reduced drastically. Moreover, the participants for whom we found poor performance for the model changed according to the sensor placement. For activity-specific scenarios, in intra-position cases, we found that the heuristic features work better for high-intensity activities. We recorded poor precision, recall, and F1-Score for low-intensity activities, such as Lying and Sitting.

Regarding the inter-position cases, we found comparatively better results when we trained the model using the data from the hand position. The worst result was when we trained the model using the data from the backpack position. One interesting finding is that the model that we trained using the data from the hand position, which encompasses data of comparatively more variations because of the frequent movement of the hand, showed the highest accuracy when using data from backpack or pocket position as test data. On the contrary, the model performed poorly when it was trained using the data from the backpack, which encompasses fewer data variations because of the less frequent movements of the backpack. The difference between the values of the evaluation metrics for intra-position and inter-position cases was approximately 10%. The accuracies for the inter-position cases were around 65%. The model performance was informative, considering we used data from one accelerometer and simple heuristic features. The heuristics features were particularly proposed for solving the orientation problem, and we wanted to find out the heuristic features’ effectiveness in the placement problem. The performance of the heuristic features in inter-position cases indicates that if we fuse the heuristic features with other proposed approaches to solve the sensor-placement problem, then there is a high chance that the performance will increase. In addition, for both intra-position and inter-position cases, if we use other types of sensors, such as a Gyroscope and Magnetometer along with an accelerometer, we may find better results using the heuristic features.

We had some interesting findings regarding the participant-specific and activity-specific scenarios for inter-position cases. We observed that, in some cases, when we had good accuracy for a particular subject’s data from one specific position, we had low accuracy for that same subject’s data from a different position. Such a case was for Participant 22 when the model was trained using the data from the backpack position and tested using the data from the other two positions. Similar to the intra-position scenario, in inter-position scenarios, we observed that the models performed poorly for some participants in every inter-position case, reducing the average accuracy for all cases. In the activity-specific scenario, the findings’ pattern was similar to those for intra-position cases. The heuristic features could not perform well for low-intensity activities, but the result was good for high-intensity activities, especially for Walking. However, among the high-intensity activities, the performance for Running at 5 METs was unsatisfactory.

In summary, the performance of the heuristic features with 1D-CNN–LSTM was promising in both intra-position and inter-position cases. We used data from only one accelerometer and performed a Leave-10-Subject-Out Cross-Validation approach. We tried to replicate a practical scenario for a machine learning model and evaluate the performance of the heuristic feature in such cases. For inter-position cases, using other types of sensors might help. An interesting future study would be to observe how the heuristic features perform if fused with existing or newly collected data designed to solve the sensor placement problem.

Our study had research gaps we would like to explore in future work. We explored the effectiveness of the heuristic features using one type of model. Several other classifiers could be promising, and we will investigate other classifiers’ performance on the same data set in future work. In addition, we have not investigated the effectiveness of time and frequency-domain features on our data set. In the future, we will evaluate the performance of time and frequency domain features and compare the results with those achieved using heuristic features. For our data set, we used signals from a single smartphone accelerometer. We should explore how the results change by including signals from the gyroscope and other smartphone sensors. We can fuse the heuristic features with other proposed techniques for solving the sensor placement problem and evaluate its effectiveness for inter-position cases. We only conducted our study for six activities. In future, we intend to conduct the same study with more activities and variations. However, we think that exploring the effectiveness of the heuristic features for different positions in a practical manner would help other studies have a proper idea about the potency of the heuristic features and develop accuracy using other techniques with these heuristic features.

## Conclusion

This study examined whether simple heuristic features could help solve the sensor orientation and placement problems when conducting HAR using a single smartphone accelerometer. Our study used the 1D-CNN–LSTM classifier as it utilizes both CNN’s feature extraction power and LSTM’s information-persisting ability. Our study concludes that the heuristic features adequately solve the sensor orientation problem despite a simple study protocol. We found the best accuracy (73.64%) in solving the sensor orientation problem using the heuristic features when the smartphone was placed in the backpack. When working with sensor placement issues, we acquired the best accuracy (68.66%) when we trained the model using the data from position: hand and tested using the data from position: backpack. In addition, we found the heuristic features to be more effective for high-intensity activities. In future, we want to perform the same study using other machine learning algorithms and present a comparative analysis. Furthermore, we will be fusing other methods that eliminate sensor orientation and placement problems with heuristic features to investigate if the outcome can be improved further or not. We believe that the findings from our study will help other researchers decide how to approach solving sensor orientation and placement problems when using heuristic features. Finally, we hope that the outcome of this study will assist in building a robust HAR model in terms of sensor orientation and position variation in the future.

## Methods

In this section, we will first discuss the data accumulation process. Following, we will explain the data pre-processing and feature extraction procedure. Then, we will briefly discuss the feature selection approach and describe the 1D-CNN–LSTM architecture we used.

### Data accumulation

For our study, we collected data from 42 healthy participants for six different activities with varying intensities: Lying, Sitting, Walking, Running at 3 METs, Running at 5 METs, and Running at 7 METs. We acquired ethical approval from the Memorial University Interdisciplinary Committee on Ethics in Human Research (ICEHR #20180188-EX). Before commencing the data accumulation procedure, each participant had to complete the Physical Activity Readiness Questionnaire (PAR-Q). There were 18 male and 24 female participants. The average age, height and weight were 29 (range = 18–56 years) years, 169.17 cm (range = 143–185 cm) and 68.19 kg (range = 43–95.2 kg), respectively. Each participant performed nine trials to complete the data collection protocol. While performing the trials, the participant carried three Samsung Galaxy S7 smartphones (SM-G930W8) in three locations. The locations were the participant’s right pocket, backpack and right hand. The data accumulation process was 65 min long. The order of the trials with duration is given in Table 4. Trial 1 is the trial with which the participants started the data collection protocol, and Trial 9 refers to the last trial to be completed.

**Table 4:** Trial order and duration of data collection protocol

Trial order	Activity	Duration (min)
1	Lying down	5
2	Sitting	5
3	Walking	10
4	Lying down	5
5	Running at 3 METs	10
6	Lying down	5
7	Running at 5 METs	10
8	Sitting	5
9	Running at 7 METs	10

An android application called Ethica Data [67] was used to collect sensor data. The application recorded the data from the accelerometer sensor’s *X*, *Y* and *Z* axes embedded into the smartphone. The application continuously recorded the sensor’s value and uploaded the value to the server. During the data collection, the participants were free to keep the smartphone in any arbitrary orientation. We collected the data in an indoor environment. We used a treadmill to accumulate data for walking and running at three speeds. We used the Metabolic Equivalent of Task (MET) to quantify the running intensities or speeds. METs are a ratio of the oxygen consumption rate of a person to the corresponding person’s weight. We preferred MET to walk speed, cadence or stride length to measure the intensity, because those units are prone to generate different expenditures for different persons. We wanted to ensure that the participants performed the activity with the same intensity. The mathematical equation to define the MET is given in (1):

1 $${\text{MET}} = \frac{{{\text{Oxygen}}\;{\text{Consumption}}\;{\text{Rate}} \;\left( {\frac{{{\text{milileter}}}}{{{\text{minute}}}}} \right)}}{{3.5 \times {\text{weight}}\; \left( {{\text{kg}}} \right)}}.$$

We selected these particular activities in our study to ensure the presence of the most common daily activities. Besides, few HAR studies combined activity types and activity intensity recognition in their work.

### Data pre-processing

We performed data resampling and data imputation on the data set. The optimization technique of the Ethica App did not let the app maintain the same data uploading frequency. As a result, the frequency ranged from 5 to 19 Hz. We upsampled the data set to a constant frequency of 30 Hz to eliminate this data imbalance using a published method [68]. Another challenge with the data was missing values. Missing data occurred because of the temporal connection loss between the Ethica App and the server. We used linear data imputation to impute missing values. The number of samples for each activity at each position after pre-processing is shown in Table 5,

**Table 5:** Number of samples for each activity class at each position after pre-processing

Position: pocket		Position: backpack		Position: hand	
Activity name	Sample count	Activity name	Sample count	Activity name	Sample count
Sitting	879,325	Sitting	879,612	Sitting	879,855
Lying	1,266,292	Lying	1,268,078	Lying	1,269,428
Walking	765,246	Walking	765,375	Walking	765,266
Running at 3 METS	984,997	Running at 3 METS	986,512	Running at 3 METS	986,310
Running at 5 METS	986,446	Running at 5 METS	986,064	Running at 5 METS	985,187
Running at 7 METS	992,654	Running at 7 METS	995,726	Running at 7 METS	992,154

### Feature extraction

**Fig. 8:**
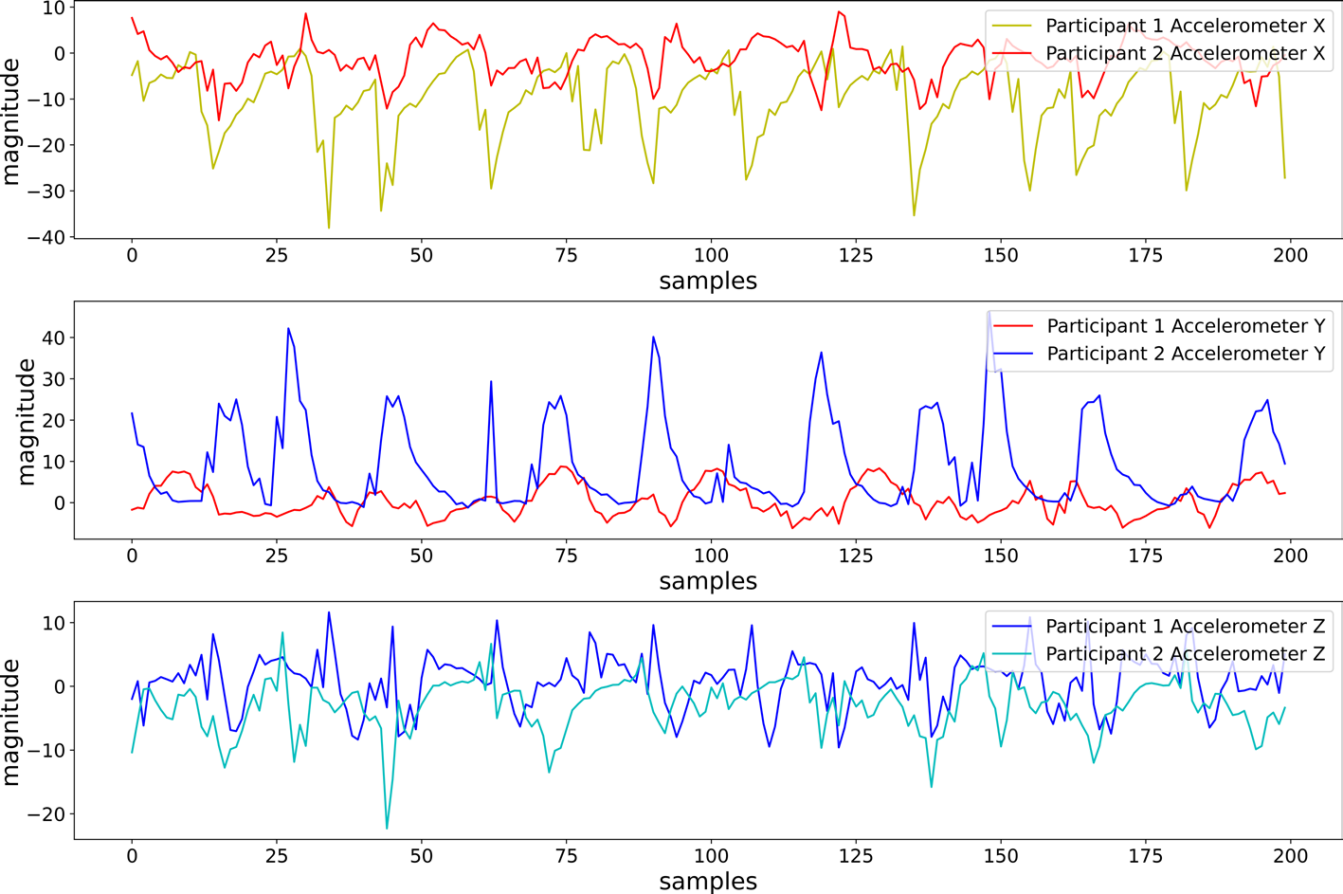
Accelerometer axis for different participants performing running at 7 METs (position: backpack)

We extracted 9 orientation-invariant heuristic features using the formula [26] to address the orientational dependency problem. Since the participants had the freedom to place the smartphones in the pre-determined position in any orientation, we experienced different ranges and patterns in sensor values for different participants, even though they were performing the same activity. Figure 8 shows the differences in patterns and ranges of accelerometer axes due to the sensor orientation, while different participants performed the same activity (Running at 7 METs), keeping the smartphone in their backpacks.

We extracted the previously introduced 9 orientation invariant heuristic features to eliminate this problem. The formulas to extract the 9 orientation-invariant heuristic features are given below,

2 $$w_{1} \left[ n \right] = \left\| {\overrightarrow {{v_{n} }} } \right\| ,$$

3 $$w_{2} \left[ n \right] = \left\| {\Delta \overrightarrow {{v_{n} }} } \right\|,$$

4 $$w_{3} \left[ n \right] = \left\| {\Delta^{2} \overrightarrow {{v_{n} }} } \right\| ,$$

5 $$w_{4} \left[ n \right] = \angle \left( {\overrightarrow {{v_{n} }} , \overrightarrow {{v_{n + 1} }} } \right),$$

6 $$w_{5} \left[ n \right] = \angle \left( {\Delta \overrightarrow {{v_{n} }} , \Delta \overrightarrow {{v_{n + 1} }} } \right),$$

7 $$w_{6} \left[ n \right] = \angle \left( {\Delta^{2} \overrightarrow {{v_{n} }} , \Delta^{2} \overrightarrow {{v_{n + 1} }} } \right),$$

8 $$w_{7} \left[ n \right] = \angle \left( {\overrightarrow {{p_{n} }} , \overrightarrow {{p_{n + 1} }} } \right)\;{\text{where}}\;\overrightarrow {{p_{n} }} = \overrightarrow {{v_{n} }} \times \overrightarrow {{v_{n + 1} }} ,$$

9 $$w_{8} \left[ n \right] = \angle \left( {\overrightarrow {{q_{n} }} , \overrightarrow {{q_{n + 1} }} } \right)\;{\text{where}}\;\overrightarrow {{q_{n} }} = \Delta \overrightarrow {{v_{n} }} \times \Delta \overrightarrow {{v_{n + 1} }} ,$$

10 $$w_{9} \left[ n \right] = \angle \left( {\overrightarrow {{r_{n} }} , \overrightarrow {{r_{n + 1} }} } \right)\;{\text{where}}\;\overrightarrow {{r_{n} }} = \Delta^{2} \overrightarrow {{v_{n} }} \times \Delta^{2} \overrightarrow {{v_{n + 1} }} .$$

Here,

$\overrightarrow {{v_{n} }} = \left( {v_{x} \left[ n \right], v_{y} \left[ n \right], v_{z} \left[ n \right]} \right)$ defines a vector, where $v_{x} \left[ n \right]$, $v_{y} \left[ n \right]$, $v_{z} \left[ n \right]$, were values of the accelerometer *x*-axis, *y*-axis, and *z*-axis, respectively, at any time sample *n*. $\Delta \overrightarrow {{v_{n} }} = v_{n + 1} - v_{n}$ and $\Delta^{2} \overrightarrow {{v_{n} }} = v_{n + 1} - v_{n} ,$ defined first-order and second-order time differences, respectively.

$$w_{t} = {\text{extracted}}\;{\text{heuristic}}\;{\text{features}}\;{\text{for}}\;t = 1 \;{\text{to}}\; 9,$$

$$\left\| {\vec{m}} \right\| = {\text{Euclidean}} \;{\text{norm}}\;{\text{of}}\;{\text{vector}}\;m,$$

11 $$\angle \left( {\vec{a}, \vec{b}} \right) = \cos^{ - 1} \left( {\frac{{\vec{a} \cdot \vec{b}}}{{ \left\| {\vec{a}} \right\| \left\| {\vec{b}} \right\|}}} \right) = {\text{angle}}\;{\text{between}}\;{\text{vector}}\;a\;{\text{and}}\;{\text{vector}}\;b\;{\text{where}}\;\vec{a} \cdot \vec{b}\;{\text{denotes}}\;{\text{their}}\;{\text{dot}}\;{\text{product}}.$$

A more detailed explanation of the features can be found in [26]. Although they introduced the nine features mentioned and used them to eliminate the orientational effect, in a previous study [69], we found the first four features $w_{1} ,{ }w_{2} ,{ }w_{3,}$ and $w_{4,}$ to be the most significant and effective in reducing the orientational effect. Therefore, for our study, we only used the first four features.

#### Heuristic features for different orientations

**Fig. 9:**
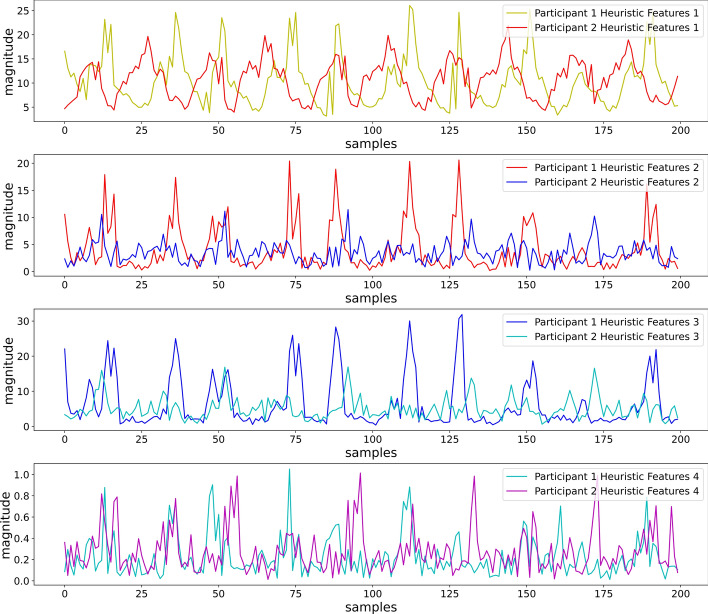
First 4 heuristic features for different participants performing running at 7 METs (position: backpack)

From Fig. 9, we can observe that the 4 heuristic features were able to introduce enough similarity for the feature values, while two different participants placed the smartphone in a backpack and performed the same activity (Running at 7 METs).

**Fig. 10:**
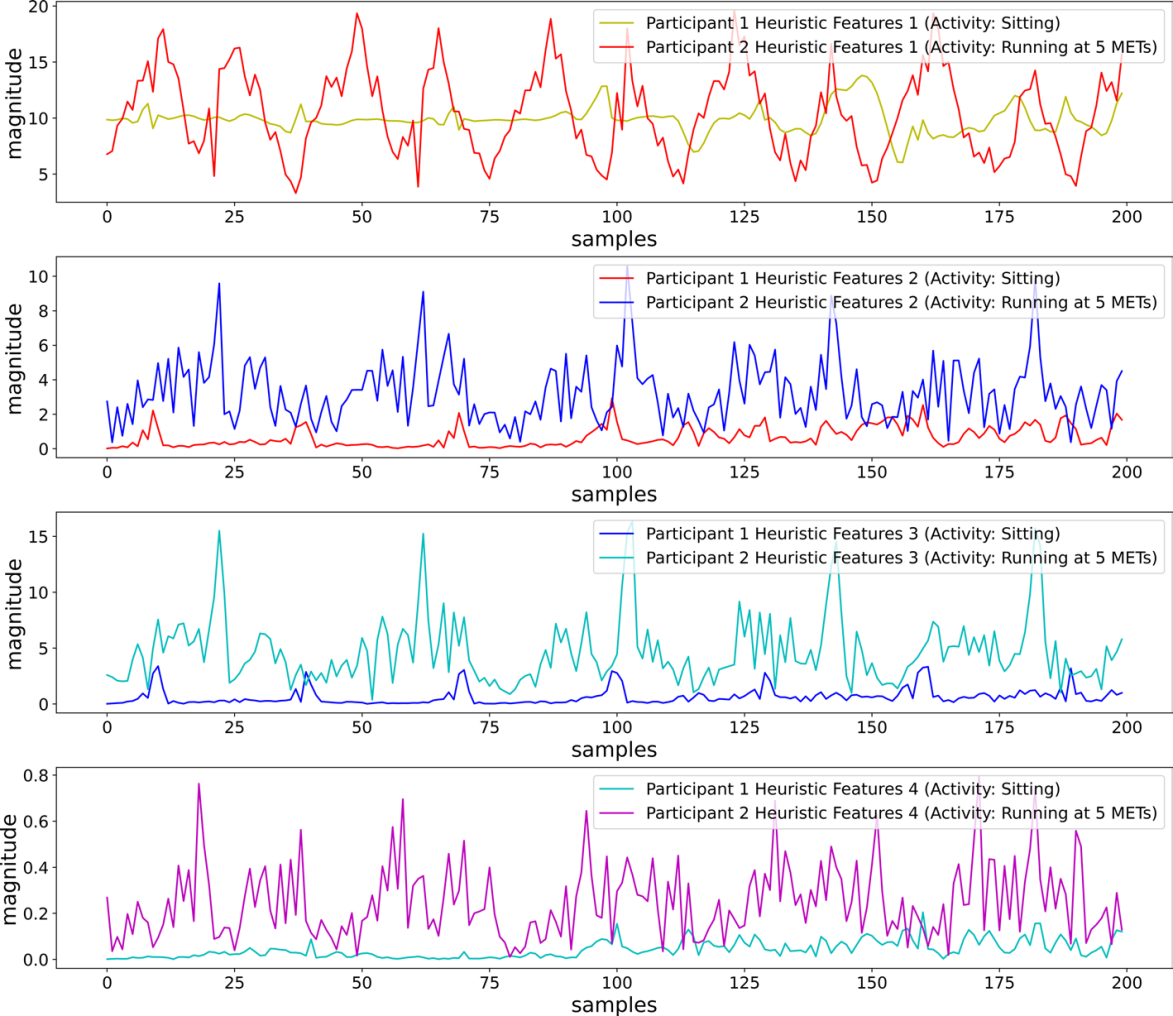
First 4 heuristic features for different participants performing sitting and running at 5 METs (position: backpack)

Visual inspection of Fig. 10 shows that features were able to maintain dissimilarity for the feature values, while two different participants placed the smartphone in a backpack and performed different activities (Sitting and Running at 5 METs). The heuristic features reduced the sensor orientation effect from the sensor values. The four features are also simple to extract, which can reduce computational complexity compared to the other feature-extracting methods with numerous features that need to be extracted to eliminate the orientational problem.

#### Heuristic features for different placements

**Fig. 11:**
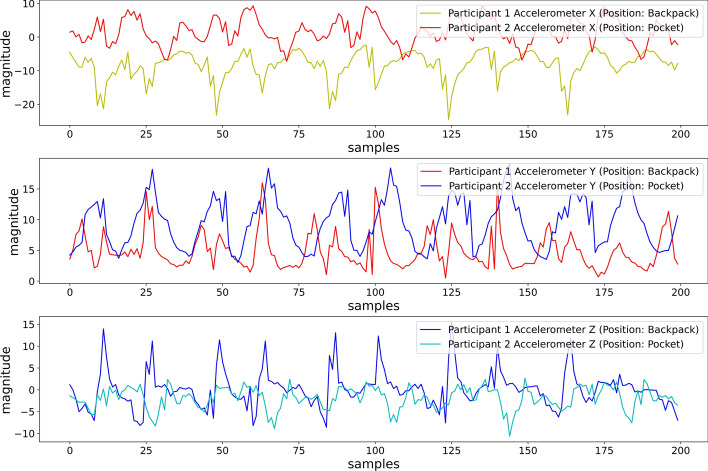
Differences in range and patterns of accelerometer axes due to different sensor placements

We investigated the patterns and ranges of the raw accelerometer values and heuristic features for different smartphone placements. The raw accelerometer values should differ in ranges and patterns for the same activity performed by different participants when keeping the smartphone in different placements. From Fig. 11, we can observe the dissimilarity in patterns and ranges of raw accelerometer values, while two participants performed Running at 5METs, keeping the smartphone in two different locations (Backpack and Pocket).

**Fig. 12:**
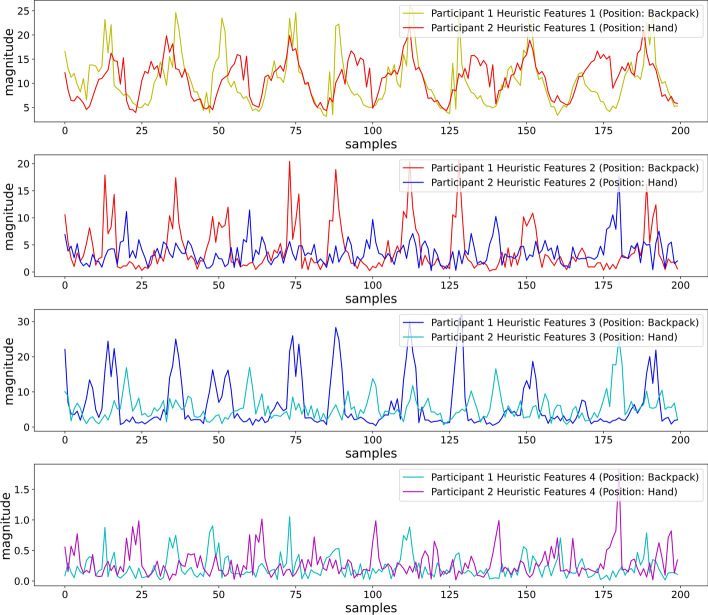
First 4 heuristic features for different smartphone placements and different participants running at 5 METs

From Fig. 12, we can observe that the first heuristic feature showed similarities in the values and patterns, while the two participants performed the same activity, Running at 5 METs, by keeping the smartphones in different locations (Backpack and Pocket). For the remaining 3 heuristic features, the similarities in ranges looked promising, but the patterns differed substantially.

**Fig. 13:**
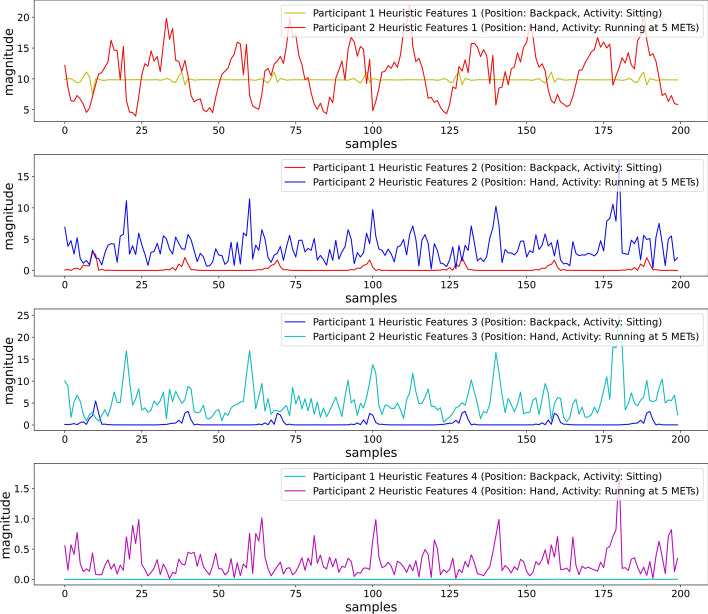
First 4 heuristic features for different smartphone placements and different participants performing different activities

Besides, the heuristic features could maintain the dissimilarity in the feature values and range for different activities (Sitting and Running at 5 METs) performed by different participants, keeping the smartphone in different locations (Backpack and Pocket), as depicted in Fig. 13.

### 1D-CNN–LSTM architecture

In this section, we will discuss our deep-learning approach. Although the heuristic features tried to reduce the gap between the sensor values for the same activity in different placements (i.e., hand, pocket or backpack), there were still substantial differences among the sensor values for different placements. We used a hybrid 1D-CNN–LSTM architecture to address the sensor placement problem. In our proposed model architecture, there were two significant parts. The first part contained the CNN model, and the second included the LSTM and fully connected layers. The reason behind using CNN was its automatic feature extraction capability. In general, a CNN model takes images or data matrices as input. The convolution layer of CNN applies multiple filters or kernels on the feed images or data matrices and extracts meaningful feature maps. The number of feature maps depends on the number of filters used. If $n$ number of filters are applied on a single data matrix, then we will get $n$ number of feature maps, where each feature map will try to extract a distinctive feature for that data matrix. After extracting the feature maps, CNN uses the pooling layer to reduce the size of the feature maps. The average or max pooling layer reduces the feature map’s size. The feature maps can be regarded as the automatically extracted features for the input data matrices. In the convolution layer, we propagate the kernels or filters on the data matrices in two different ways. If we propagate the filters in two directions at a time, we call the model 2D-CNN or conventional CNN. If we propagate the filters in only one direction, we call it 1D-CNN. In general, we use 2D-CNN for images and 1D-CNN for data matrices. As mentioned, CNN can be combined with LSTM to maintain temporal and spatial dependency. In an LSTM model, there can be one or more LSTM layers. Each LSTM layer contains multiple LSTM cells, and each LSTM cell has three gates: Forget gate, Input gate and Output gate. We need to feed data matrices as input to the LSTM model. If a data matrix has *n* samples, then we can denote the samples as $t_{i}$, where $i = 1,2,3 \ldots n$. When the Input gate processes any particular sample $t_{i}$, the Forget gate decides the information to preserve from the previous sample $t_{i - 1}$. The Output gate then combines the information from the Input gate and Forget gate to predict the current sequence or data matrix. If an LSTM model follows a CNN model, then the feature maps generated by the CNN model act as input to the LSTM model. Using the extracted feature maps of the CNN model, the LSTM model can find better temporal dependency for a sequence or data matrix. The output from the LSTM model goes to the fully connected layers made of conventional neurons to make the final prediction. We assumed that the CNN portion of the proposed architecture would be capable of bringing meaningful feature maps, which will help reduce the similarity gap in heuristic feature values observed in the case of non-identical placements of smartphones.

Our proposed 1D-CNN–LSTM architecture was designed as a classification model for classifying human activities. The 1D-CNN–LSTM model contained six convolution layers with 512, 256, 64, 128, 256, and 512 filters, followed by an LSTM layer with 512 LSTM cells. Then, we added four fully connected layers with 100, 28, 64 and 6 neurons. We had average pooling layers after the first, third and final convolution layers with a pool size of 3. We also introduced some dropout layers to reduce the overfitting issue in our model. A more detailed description of the model is depicted in Table 6. We used an “Adam” optimizer with a learning rate of 0.0001. We determined all the hyperparameters for our deep learning algorithm using the trial and error method.

**Table 6:** Architecture of the 1D-CNN-LSTM model

Parts of architecture	Components of each part						
	Layer’s name	Number of filters	Kernel size	Pool size	Activation function	Padding type	Dropout ratio
CNN	Convolution	512	5		relu	Same	
	Dropout						0.3
	Average pooling			3		Same	
	Convolution	256	3		relu	Same	
	Dropout						0.3
	Convolution	64	3		relu	Same	
	Average pooling			3		Same	
	Convolution	128	3		relu	Same	
	Convolution	256	5		relu	Same	
	Dropout						0.3
	Convolution	512	7		relu	Same	
	Dropout						0.3
	Average pooling			3		Same	

	Layer’s name			Number of units			Activation function
LSTM	LSTM			512			tanh

	Layer’s name			Number of neurons			Activation function
Fully connected network	Dense			100			relu
	Dense			28			relu
	Dense			64			relu
	Dense			6			softmax

### Validation procedure

There were data from 42 participants. We used 30 participants’ data in the training phase, 10 for testing and 2 for validation in each iteration of our validation procedure. The participant’s data in the validation set were constant, but the training and test data changed as we used Leave-*N*-Subject-Out Cross-Validation. In our case, the value of *N* was 10, which made our procedure a Leave-10-Subject-Out Cross-Validation technique. As we had data from 40 participants for the training and testing phase, 4 iterations were required for the whole validation procedure. We had 10 different participants’ data in the test set at each iteration. As mentioned, we decided to inspect two separate scenarios: intra-position (i.e., sensor orientation problem) and inter-position (i.e., sensor placement problem) evaluations. In the intra-position evaluation, the 1D-CNN–LSTM model was trained and tested using the data from the same position. In the inter-position scenario, the model was trained using data from one position but tested using the data from the other two positions. To accomplish the intra-position and inter-position evaluation, we trained the 1D-CNN–LSTM model for a particular position using the heuristic features for the 30 participants in the training set. Then, we computed the evaluation metrics using the data of 10 participants in the test set for all three positions. For instance, if the model was trained using the heuristic features of 30 participants in the training set for the pocket position, then we computed the evaluation metrics using the heuristic features of 10 participants in the test set for all three positions: pocket, hand and backpack. In this manner, both intra-position and inter-position results were accumulated for all three positions. According to our validation approach, we had to train the model 4 times to follow the Leave-10-Out Cross-Validation technique for each position. Since we were conducting our study for 3 different positions, we needed to train the model 12 times. We used an early stopping technique in the training of the 1D-CNN–LSTM model. The early stopping technique was designed, so that the model would stop training if the model’s accuracy for the validation data did not improve within the successive 20 epochs. The 1D-CNN–LSTM model needed the training and test data to be segmented into data matrices or windows, because 1D-CNN–LSTM works with data windows or data matrices. We segmented the training and test data in each iteration using a window length of 65 samples with an overlapping ratio of 98.46%. That means each window had 65 samples, and two consecutive windows had 64 samples in common. We used the window length of 65, because we found it to be both computational and time-efficient in our previous study [69]. The information for the validation approach is organized in Table 7.

**Table 7:** Properties of the validation procedure for each model in each iteration of the Leave-10-Subject-Out Cross-Validation technique

Positions of smartphone			Number of participants		Parameter values	
Training data	Test data for intra-position validation	Test data for inter-position validation	Training set	Test set	Window size	Batch Size
Pocket	Pocket	Backpack and hand	30	10	65	2000
Backpack	Backpack	Pocket and hand	30	10	65	2000
Hand	Hand	Pocket and backpack	30	10	65	2000

## Data Availability

The data sets generated and/or analyzed during the current study are not publicly available, but we may publish them soon.

## References

[1] Barua A, Masum AKM, Bahadur EH, Alam MR, Chowdhury M, Alam MS (2020). Human activity recognition in prognosis of depression using long short-term memory approach. Int J Adv Sci Technol.

[2] Bahadur EH, Masum AKM, Barua A, Uddin MZ (2021). Active sense: early staging of non-insulin dependent diabetes mellitus (NIDDM) hinges upon recognizing daily activity pattern. Electronics.

[3] Subasi A, Radhwan M, Kurdi R, Khateeb K. IoT based mobile healthcare system for human activity recognition. In: 2018 15th learning and technology conference (L&T). IEEE; 2018. p. 29–34.

[4] Zdravevski E, Lameski P, Trajkovikj V, Pombo N, Garcia N (2018). Importance of personalized health-care models: a case study in activity recognition. Stud Health Technol Inform.

[5] Jalal A, Kamal S, Kim D (2014). A depth video sensor-based life-logging human activity recognition system for elderly care in smart indoor environments. Sensors.

[6] Zhuang Z, Xue Y (2019). Sport-related human activity detection and recognition using a smartwatch. Sensors.

[7] Yin J, Yang Q, Pan JJ (2008). Sensor-based abnormal human-activity detection. IEEE Trans Knowl Data Eng.

[8] Ha S, Choi S. Convolutional neural networks for human activity recognition using multiple accelerometer and gyroscope sensors. In: 2016 international joint conference on neural networks (IJCNN). IEEE; 2016. p. 381–8.

[9] Wang A, Chen G, Yang J, Zhao S, Chang C-Y (2016). A comparative study on human activity recognition using inertial sensors in a smartphone. IEEE Sens J.

[10] Barna A, Masum AKM, Hossain ME, Bahadur EH, Alam MS. A study on human activity recognition using gyroscope, accelerometer, temperature and humidity data. In: 2019 international conference on electrical, computer and communication engineering (ECCE). IEEE; 2019. p. 1–6.

[11] Attal F, Mohammed S, Dedabrishvili M, Chamroukhi F, Oukhellou L, Amirat Y (2015). Physical human activity recognition using wearable sensors. Sensors.

[12] Olguın DO, Pentland AS. Human activity recognition: accuracy across common locations for wearable sensors. In: Proceedings of 2006 10th IEEE international symposium on wearable computers, Montreux, Switzerland. Citeseer; 2006. p. 11–14.

[13] Zhang M, Sawchuk A. A feature selection-based framework for human activity recognition using wearable multimodal sensors. In: 6th international ICST conference on body area networks. 2012.

[14] Masum AKM, Barua A, Bahadur EH, Alam MR, Chowdhury MAUZ, Alam MS. Human activity recognition using multiple smartphone sensors. In: 2018 international conference on innovations in science, engineering and technology (ICISET). IEEE; 2018. p. 468–73.

[15] Hassan MM, Uddin MZ, Mohamed A, Almogren A (2018). A robust human activity recognition system using smartphone sensors and deep learning. Futur Gener Comput Syst.

[16] Ronao CA, Cho S-B (2016). Human activity recognition with smartphone sensors using deep learning neural networks. Expert Syst Appl.

[17] Ronao CA, Cho S-B. Deep convolutional neural networks for human activity recognition with smartphone sensors. In: International conference on neural information processing. Springer; 2015. p. 46–53.

[18] Khan AM, Lee Y-K, Lee S-Y, Kim T-S. Human activity recognition via an accelerometer-enabled-smartphone using kernel discriminant analysis. In: 2010 5th international conference on future information technology. IEEE; 2010. p. 1–6.

[19] Filippoupolitis A, Oliff W, Takand B, Loukas G (2017). Location-enhanced activity recognition in indoor environments using off the shelf smart watch technology and BLE beacons. Sensors.

[20] Shoaib M, Incel OD, Scholten H, Havinga P. Smokesense: online activity recognition framework on smartwatches. In: International conference on mobile computing, applications, and services. Springer; 2018. p. 106–24.

[21] Knighten J, McMillan S, Chambers T, Payton J. Recognizing social gestures with a wrist-worn smartband. In: 2015 IEEE international conference on pervasive computing and communication workshops (PerCom workshops). IEEE; 2015. p. 544–9.

[22] Atallah L, Lo B, King R, Yang G-Z. Sensor placement for activity detection using wearable accelerometers. In: 2010 International conference on body sensor networks. IEEE; 2010. p. 24–9.

[23] Davoudi A, Mardini MT, Nelson D, Albinali F, Ranka S, Rashidi P, Manini TM (2021). The effect of sensor placement and number on physical activity recognition and energy expenditure estimation in older adults: validation study. JMIR Mhealth Uhealth.

[24] Yang R, Wang B (2016). PACP: a position-independent activity recognition method using smartphone sensors. Information.

[25] Chen Y, Shen C (2017). Performance analysis of smartphone-sensor behavior for human activity recognition. Ieee Access.

[26] Yurtman A, Barshan B (2017). Activity recognition invariant to sensor orientation with wearable motion sensors. Sensors.

[27] Rasekh A, Chen C-A, Lu Y. Human activity recognition using smartphone. arXiv preprint. 2014. arXiv:1401.8212.

[28] Alo UR, Nweke HF, Teh YW, Murtaza G (2020). Smartphone motion sensor-based complex human activity identification using deep stacked autoencoder algorithm for enhanced smart healthcare system. Sensors.

[29] Siirtola P, Röning J. User-independent human activity recognition using a mobile phone: offline recognition vs. real-time on device recognition. In: Distributed computing and artificial intelligence: 9th international conference. Springer; 2012. p. 617–27.

[30] Zdravevski E, Lameski P, Trajkovik V, Kulakov A, Chorbev I, Goleva R, Pombo N, Garcia N (2017). Improving activity recognition accuracy in ambient-assisted living systems by automated feature engineering. IEEE Access.

[31] Guo H, Chen L, Chen G, Lv M (2016). Smartphone-based activity recognition independent of device orientation and placement. Int J Commun Syst.

[32] Chen Z, Zhu Q, Soh YC, Zhang L (2017). Robust human activity recognition using smartphone sensors via CT-PCA and online SVM. IEEE Trans Ind Inf.

[33] Ustev YE, Durmaz Incel O, Ersoy C. User, device and orientation independent human activity recognition on mobile phones: challenges and a proposal. In: Proceedings of the 2013 ACM conference on pervasive and ubiquitous computing adjunct publication. 2013. p. 1427–36.

[34] Anguita D, Ghio A, Oneto L, Parra X, Reyes-Ortiz JL. Human activity recognition on smartphones using a multiclass hardware-friendly support vector machine. In: Ambient assisted living and home care: 4th international workshop, IWAAL 2012, Vitoria-Gasteiz, Spain, December 3–5, 2012 proceedings 4. Springer; 2012. p. 216–23.

[35] Tran DN, Phan DD. Human activities recognition in android smartphone using support vector machine. In: 2016 7th international conference on intelligent systems, modelling and simulation (ISMS). IEEE; 2016. p. 64–8.

[36] Anguita D, Ghio A, Oneto L, Parra X, Reyes-Ortiz JL. Training computationally efficient smartphone–based human activity recognition models. In: Artificial neural networks and machine learning—ICANN 2013: 23rd international conference on artificial neural networks Sofia, Bulgaria, September 10–13, 2013 proceedings 23. Springer; 2013. p. 426–33.

[37] Bayat A, Pomplun M, Tran DA (2014). A study on human activity recognition using accelerometer data from smartphones. Procedia Comput Sci.

[38] Uddin MT, Uddiny MA. Human activity recognition from wearable sensors using extremely randomized trees. In: 2015 international conference on electrical engineering and information communication technology (ICEEICT). IEEE; 2015. p. 1–6.

[39] Gupta S, Kumar A (2015). Human activity recognition through smartphone’s tri-axial accelerometer using time domain wave analysis and machine learning. Int J Comput Appl.

[40] Paul P, George T. An effective approach for human activity recognition on smartphone. In: 2015 IEEE international conference on engineering and technology (ICETECH). IEEE; 2015. p. 1–3.

[41] Kaghyan S, Sarukhanyan H (2012). Activity recognition using k-nearest neighbor algorithm on smartphone with tri-axial accelerometer. Int J Inform Models Anal THEA Int Sci Soc Bulg.

[42] Fan L, Wang Z, Wang H. Human activity recognition model based on decision tree. In: 2013 international conference on advanced cloud and big data. IEEE; 2013. p. 64–8.

[43] Lara OD, Labrador MA. A mobile platform for real-time human activity recognition. In: 2012 IEEE consumer communications and networking conference (CCNC). IEEE; 2012. p. 667–1.

[44] Yang J, Nguyen MN, San PP, Li XL, Krishnaswamy S. Deep convolutional neural networks on multichannel time series for human activity recognition. In: Twenty-fourth international joint conference on artificial intelligence. 2015.

[45] Zeng M, Nguyen LT, Yu B, Mengshoel OJ, Zhu J, Wu P, Zhang J. Convolutional neural networks for human activity recognition using mobile sensors. In: 6th international conference on mobile computing, applications and services. IEEE; 2014. p. 197–205.

[46] Xu Y, Qiu TT (2021). Human activity recognition and embedded application based on convolutional neural network. J Artif Intell Technol.

[47] Wan S, Qi L, Xu X, Tong C, Gu Z (2020). Deep learning models for real-time human activity recognition with smartphones. Mobile Netw Appl.

[48] Varshney N, Bakariya B, Kushwaha AKS, Khare M (2022). Human activity recognition by combining external features with accelerometer sensor data using deep learning network model. Multimed Tools Appl.

[49] Almaslukh B, Al Muhtadi J, Artoli AM (2018). A robust convolutional neural network for online smartphone-based human activity recognition. J Intell Fuzzy Syst.

[50] Inoue M, Inoue S, Nishida T (2018). Deep recurrent neural network for mobile human activity recognition with high throughput. Artif Life Robot.

[51] Alessandrini M, Biagetti G, Crippa P, Falaschetti L, Turchetti C (2021). Recurrent neural network for human activity recognition in embedded systems using PPG and accelerometer data. Electronics.

[52] Murad A, Pyun J-Y (2017). Deep recurrent neural networks for human activity recognition. Sensors.

[53] Xia K, Huang J, Wang H (2020). LSTM-CNN architecture for human activity recognition. IEEE Access.

[54] Mekruksavanich S, Jitpattanakul A. Smartwatch-based human activity recognition using hybrid LSTM network. In: 2020 IEEE sensors. IEEE; 2020. p. 1–4.

[55] Mekruksavanich S, Jitpattanakul A (2021). LSTM networks using smartphone data for sensor-based human activity recognition in smart homes. Sensors.

[56] Muhammad K, Ullah A, Imran AS, Sajjad M, Kiran MS, Sannino G, de Albuquerque VHC (2021). Human action recognition using attention based LSTM network with dilated CNN features. Futur Gener Comput Syst.

[57] Deep S, Zheng X. Hybrid model featuring CNN and LSTM architecture for human activity recognition on smartphone sensor data. In: 2019 20th international conference on parallel and distributed computing, applications and technologies (PDCAT). IEEE; 2019. p. 259–64.

[58] Khatun MA, Yousuf MA, Ahmed S, Uddin MZ, Alyami SA, Al-Ashhab S, Akhdar HF, Khan A, Azad A, Moni MA (2022). Deep CNN-LSTM with self-attention model for human activity recognition using wearable sensor. IEEE J Transl Eng Health Med.

[59] Challa SK, Kumar A, Semwal VB (2021). A multibranch CNN-BiLSTM model for human activity recognition using wearable sensor data. Vis Comput.

[60] Tharwat A (2020). Classification assessment methods. Appl Comput Inform.

[61] Sasaki Y (2007). The truth of the F-measure. Teach Tutor Mater.

[62] Barshan B, Yüksek MC (2014). Recognizing daily and sports activities in two open source machine learning environments using body-worn sensor units. Comput J.

[63] Ugulino W, Cardador D, Vega K, Velloso E, Milidiú R, Fuks H. Wearable computing: accelerometers’ data classification of body postures and movements. In: Brazilian symposium on artificial intelligence. Springer; 2012. p. 52–61.

[64] Anguita D, Ghio A, Oneto L, Parra Perez X, Reyes Ortiz JL. A public domain dataset for human activity recognition using smartphones. In: Proceedings of the 21th international European symposium on artificial neural networks, computational intelligence and machine learning. 2013. p. 437–42.

[65] Zhang M, Sawchuk AA. USC-HAD: a daily activity dataset for ubiquitous activity recognition using wearable sensors. In: Proceedings of the 2012 ACM conference on ubiquitous computing. 2012. p. 1036–43.

[66] Casale P, Pujol O, Radeva P. Human activity recognition from accelerometer data using a wearable device. In: Iberian conference on pattern recognition and image analysis. Springer; 2011. p. 289–96.

[67] Data E. Ethica [Mobile app]. 2020.

[68] Smith J, Gossett P. A flexible sampling-rate conversion method. In: ICASSP'84 IEEE international conference on acoustics, speech, and signal processing. IEEE; 1984. p. 112–5.

[69] Barua A, Fuller D, Musa S, Jiang X (2022). Exploring orientation invariant heuristic features with variant window length of 1D-CNN-LSTM in human activity recognition. Biosensors.

